# Voluntary muscle coactivation in quiet standing elicits reciprocal rather than coactive agonist-antagonist control of reactive balance

**DOI:** 10.1152/jn.00458.2022

**Published:** 2023-05-10

**Authors:** Giovanni Martino, Owen N. Beck, Lena H. Ting

**Affiliations:** ^1^Wallace H. Coulter Department of Biomedical Engineering, Emory University and Georgia Tech, Atlanta, Georgia, United States; ^2^Department of Biomedical Sciences, University of Padova, Padua, Italy; ^3^Department of Kinesiology and Health Education, University of Texas at Austin, Austin, Texas, United States; ^4^Division of Physical Therapy, Department of Rehabilitation Medicine, Emory University, Atlanta, Georgia, United States

**Keywords:** coactivation, EMG, feedback response, postural perturbations, reciprocal activation

## Abstract

Muscle coactivation increases in challenging balance conditions as well as with advanced age and mobility impairments. Increased muscle coactivation can occur both in anticipation of (feedforward) and in reaction to (feedback) perturbations, however, the causal relationship between feedforward and feedback muscle coactivation remains elusive. Here, we hypothesized that feedforward muscle coactivation would increase both the body’s initial mechanical resistance due to muscle intrinsic properties and the later feedback-mediated muscle coactivation in response to postural perturbations. Young adults voluntarily increased leg muscle coactivation using visual biofeedback before support-surface perturbations. In contrast to our hypothesis, feedforward muscle coactivation did not increase the body’s initial intrinsic resistance to perturbations, nor did it increase feedback muscle coactivation. Rather, perturbations with feedforward muscle coactivation elicited a medium- to long-latency increase of feedback-mediated agonist activity but a decrease of feedback-mediated antagonist activity. This reciprocal rather than coactivation effect on ankle agonist and antagonist muscles enabled faster reactive ankle torque generation, reduced ankle dorsiflexion, and reduced center of mass (CoM) motion. We conclude that in young adults, voluntary feedforward muscle coactivation can be independently modulated with respect to feedback-mediated muscle coactivation. Furthermore, our findings suggest feedforward muscle coactivation may be useful for enabling quicker joint torque generation through reciprocal, rather than coactivated, agonist-antagonist feedback muscle activity. As such our results suggest that behavioral context is critical to whether muscle coactivation functions to increase agility versus stability.

**NEW & NOTEWORTHY** Feedforward and feedback muscle coactivation are commonly observed in older and mobility impaired adults and are considered strategies to improve stability by increasing body stiffness prior to and in response to perturbations. In young adults, voluntary feedforward coactivation does not necessarily increase feedback coactivation in response to perturbations. Instead, feedforward coactivation enabled faster ankle torques through reciprocal agonist-antagonist muscle activity. As such, coactivation may promote either agility or stability depending on the behavioral context.

## Graphical abstract

**Figure:**
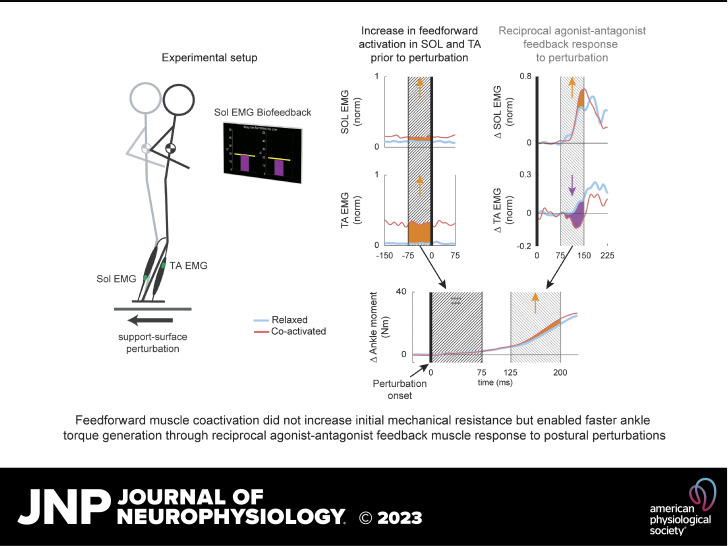

## INTRODUCTION

Patterns of coactivation between agonist and antagonist muscles can be observed in both anticipatory (feedforward) and reactive (feedback) responses to a balance perturbation, but their mutual association and functional implications are unclear. People typically increase feedforward muscle coactivation to improve their balance under challenging or threatening conditions (1–6). Following a disturbance, feedback muscle activity is elicited in response to characteristics of the perturbed kinematics (7). Due to musculoskeletal mechanics (8, 9), muscle coactivation may occur as part of the normal perturbation response depending on the type and direction of the perturbation (10–12). However, additional feedback-mediated muscle coactivation may be further elicited during particularly challenging or unpredictable conditions (12–15). Concurrent patterns of feedforward and feedback muscle coactivation are frequently observed in aging and movement impairments (e.g., spinal cord injury, Parkinson’s disease, cerebellar ataxia, spastic paraparesis) and are associated with poor balance (16–23). However, there is currently no consensus on whether feedforward and feedback coactivation are independent neural mechanisms, and whether coactivation improves or impedes balance control.

Feedforward coactivation may cause feedback coactivation, but this idea has not been directly tested during reactive balance. Previous studies have shown that tonic muscle coactivation may overwrite spinal reciprocal inhibition (24, 25), potentially facilitating antagonist muscle activity in response to a perturbation. Further evidence comes from the increased shortening reaction with increased tonic muscular activity in animals (26, 27) and humans (24, 28, 29). The shortening reaction manifests as a paradoxical coactivation of the shortened antagonist muscle together with the agonist muscle during a stretch reflex. Greater antagonist activity also characterizes the motor response to balance perturbations in people with Parkinson’s disease (18, 30, 31).

Furthermore, whether increasing feedforward coactivation improves balance control or balance capacity is still debated. Coactivation is expected to increase intrinsic joint stiffness and leads to greater resistance to external forces (32, 33). Previous studies suggest that greater ankle muscle coactivation during postural responses to perturbations in older adults yielded higher ankle stiffness (34, 35). Yet, humans are not nailed to the ground like simple inverted pendulum models, thus it remains uncertain whether coactivation and stiffening of the ankles affect the body’s resistance to a postural perturbation (36). Moreover, although ankle muscles coactivation might be beneficial when the body is translated and the ankle joint needs to be restored to its initial configuration, ankle muscle coactivation can be detrimental during a rotational perturbation where joint stiffness may further increase the center of mass (CoM) displacement due to the perturbation (37–42). Increased coactivation of ankle muscles may also affect proximal muscles through spinal reflex pathways or by changing how the initial forces of the perturbation are transmitted to proximal joints (43–45). In addition, a simulation study has shown that increased joint stiffness could reduce the body’s gain margin, defined as the distance from the critically stable feedback gain where the body would become unstable and require a stepping response to maintain balance (46).

Here, we tested whether increasing feedforward coactivation causes feedback coactivation and improves balance capacity during postural perturbations. We assessed reactive balance responses to backward support surface perturbations in young healthy adults during “relaxed” and “coactivated” conditions. We hypothesized that feedforward coactivation would increase feedback muscle coactivation in reaction to postural perturbations. In addition, we hypothesized that increasing feedforward muscle coactivation would improve balance capacity by increasing the step threshold. Therefore, we controlled the level of feedforward muscle coactivation before the perturbations through electromyographic (EMG) biofeedback and quantified the changes in EMG activity of tibialis anterior (TA) and soleus (Sol) after the perturbation. To evaluate the effects of altered EMG patterns on balance control, we evaluated changes in CoM kinematics and ankle kinematics and kinetics. To better understand the mechanisms driving reactive feedback responses following feedforward coactivation, we also used ultrasonography and shear wave tensiometry to relate muscle fascicle and tendon mechanics to feedforward coactivation and feedback response.

## METHODS

### Participants

Thirteen young adults participated in this research study (8 females and 5 males; means ± SD; age 26.5 ± 5.7 yr; height 1.71 ± 0.10 m; mass 68.3 ± 8.8 kg). None of the participants reported having a history of neurological or musculoskeletal disorders. All participants provided written informed consent before participation. The Institutional Review Board of Emory University approved the protocols.

### Protocol

**Figure 1.**
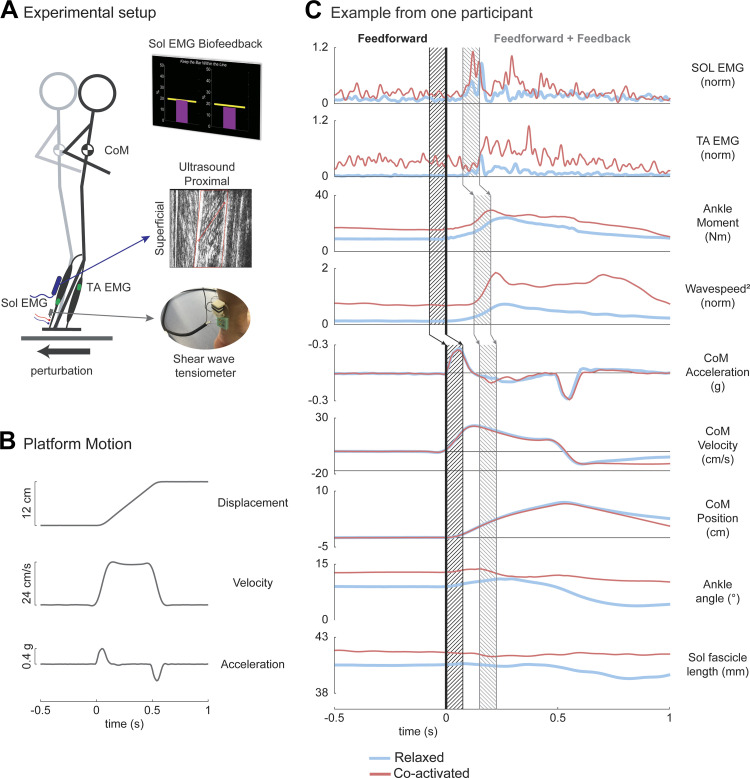
Experimental setup and example data during ramp-and-hold support surface perturbation. *A*: participants maintained standing balance throughout a series of ramp-and-hold support surface translation trials. They were instructed to either “relax,” maintaining their natural state, or “coactivate,” contracting their leg muscles so that the soleus (Sol) electromyographic (EMG) biofeedback activity achieved 250% above quiet standing activity (indicated by visual target line). A shear wave tensiometer was secured to the skin over the right Achilles tendon, and a linear-array B-mode ultrasound probe was secured to the skin over the medial gastrocnemius muscle. EMG electrodes were placed on the skin superficial to Sol and tibialis anterior (TA). Participants wore a reflective 25-marker set used by Vicon’s Plug-in Gait model to estimate center of mass (CoM) position and joint kinematics and kinetics. *B*: example of platform displacement, velocity, and acceleration traces to elicit a support-surface backward translation of 12 cm that began decelerating ∼500 ms after perturbation onset. *C*: representative signals from one participant. The effects of feedforward coactivation and feedback response were evaluated during different time windows (black diagonal lines and gray diagonal lines patterns, respectively) enclosed by the vertical lines. The tick line at *time 0* denotes the start of the perturbation.

At the beginning of each trial, we instructed participants to either “relax” or “coactivate” their leg muscles while collecting the following data: TA and Sol activity, ankle joint kinematics and kinetics, CoM kinematics, and Sol fascicle length (Fig. 1, *A*–*C*).

We instructed the participants to maintain standing balance throughout a series of ramp-and-hold support surface translation trials delivered by a custom platform (Factory Automation Systems, Atlanta, GA). Participants stood with their arms crossed about their torso and their bare feet 22 cm apart with weight evenly distributed on two force plates (AMTI, Watertown, MA), while watching a screen that displayed each leg’s Sol EMG activity (Fig. 1*A*). Approximately 2 to 7 s before each support-surface translation, we verbally instructed participants to either “relax” or “coactivate.” Upon hearing “relax,” participants stood still in their natural state. Upon hearing “coactivate,” participants attempted to maintain their joint angles and coactivate their leg muscles so that the Sol muscle activity increased 250% above that during quiet standing (indicated by visual biofeedback target line). We chose this value based on pilot experiments, to obtain a consistent and measurable effect without introducing noticeable muscle fatigue. We told participants that the biofeedback encompassed some measure of overall leg’s muscle activity, but we did not disclose that the feedback only contained Sol activation. A few participants leaned forward during the coactivation trials, introducing a potential confounding factor (47, 48). To ensure that the overall results were not affected by this leaning behavior, we excluded trials in which the initial CoM position in the coactivation conditions was located more than two times the standard deviation of the initial CoM position from the relaxed trials.

We first estimated each participant’s balance capacity by determining their step threshold during both relaxed and coactivated conditions. Step threshold was defined as the maximum backward support-surface translation (i.e., platform moved participant feet behind body/posteriorly) magnitude where participants could maintain their standing balance without taking a balance-correcting step or being caught by a safety harness (49–51). Depending on the response of the participant, we gradually increased (no step) or decreased (step) the magnitude of the platform translation starting with 15 cm. We used an adaptive method running fit (AMRF) algorithm from the Palamedes toolbox (52), which progressively reduced the change in support surface magnitude until a plateau is reached. Platform acceleration and velocity were scaled with displacement to elicit a support surface translation that began decelerating ∼500 ms after perturbation onset. Forward perturbations (i.e., platform moved participant feet ahead of body/anteriorly) were randomly interspersed with backward support-surface translations to reduce anticipatory motor adaptations (ratio 1/4). One subject did not perform the step threshold task during the coactivated condition due to a change in the protocol.

The data analyzed in this study were part of a larger data set in which we collected balance responses at different perturbation magnitudes. All participants experienced two sets of 24 ramp-and-hold support surface translations (48 trials) with 5-min seated rest preceding each set. Within each set, participants maintained standing balance during trials that moved the platform 12 cm, as well as a ∼65%, ∼75%, ∼85%, and ∼95% of their step threshold. For the current study, we only analyzed the trials in which the platform motion moved 12 cm in the backward direction with a peak velocity of 24 cm/s, to four trials for each condition [relaxed and coactivated; this was the only perturbation magnitude that was common across all participants, and it elicited primarily an ankle strategy (53)]. To mitigate adaptation to backward perturbations, participants also experienced 8 cm forward support surface translations in each block of trials. We randomized trial order within each block.

### Data Analysis

We collected ground reaction forces (AMTI, Watertown, MA) and EMG (Motion Lab Systems, Inc., Baton Rouge, LA) at 1,000 Hz, synchronized with kinematic data at 100 Hz, using a motion capture system (Vicon, Oxford, UK). Participants wore a 33-marker set according to a modified version of the Vicon’s Plug-in Gait model (7) with additional foot markers (fifth metatarsal, medial and lateral heel, medial malleolus). We calculated CoM displacement and velocity in the horizontal plane as a weighted sum of segmental masses from kinematic data, and CoM acceleration from recorded ground reaction forces divided by subject mass and platform acceleration in the horizontal plane. Ankle joint angular motion was calculated as the angle between shank and foot segments in the sagittal plane. Ankle moment was estimated from whole body kinematics and ground reaction forces using the Inverse Dynamics tool in OpenSim (54). We recorded surface EMG activity from Sol and TA on the right leg. EMG data were high-pass filtered (35 Hz, third-order zero-lag Butterworth filter), demeaned, rectified, and low-pass filtered (40 Hz) to produce a linear envelope of the signal as previously reported (55). We normalized EMG activity by the maximum value across the relaxed trials for each subject. We quantified the feedforward EMG activity of TA and Sol during a 75-ms time window before the onset of the perturbation. The feedback response was evaluated as the EMG activity during 75 to 150 ms after the perturbation onset minus the preceding (−75 to 0 ms) feedforward EMG activity. This time window was selected based on previous studies (7, 56, 57). In particular, it has been shown that the first 75-ms period of the initial burst is related to the acceleration of the perturbation (7). Furthermore, it has been shown that the relaxation time of voluntarily contracted Sol muscle in response to a visual stimulus is between 200 and 350 ms (58). Therefore, we can conservatively assume that the feedforward muscle coactivation persisted during the 75–150 ms epoch after the perturbation used to evaluate feedback responses. However, later response periods may be influenced by a decay of the voluntary feedforward activity. Similarly, the effect of feedforward and feedback activity on ankle moment and tendon force was evaluated during −75 to 0 ms and 125 to 200 ms with respect to perturbation onset. Instead, CoM and ankle kinematics, and Sol fascicle length change were evaluated during 0 to 75 ms and 150 to 225 ms after the perturbation onset.

We also collected synchronous data from shear wave tensiometer (59) and muscle ultrasound (Artus unit, Telemed, Vilnius, Lithuania). The shear wave tensiometer estimates tendon force noninvasively by tracking the propagation speed of the shear waves produced by a tapping device. The square of wave speed propagation is proportional to the force in the tendon (60). We secured the shear wave tensiometer to the skin over the Achilles tendon. Squared shear wave velocity (100 Hz) was measured by calculating the travel time between two accelerometers of a 50 Hz shear wave produced by a tapping device along the Achilles tendon. We filtered squared shear wave velocity using a 4th-order Butterworth low-pass filter (10 Hz). We normalized the wave speed to the maximum value across relaxed trials for each subject. We secured a linear-array B-mode ultrasound probe to the skin superficial of each participant’s medial gastrocnemius. We processed Sol ultrasound images using a semiautomated tracking software (61) to determine Sol fascicle length. For semiautomated images that did not accurately track the intended Sol fascicle length, we manually redefined the respective fascicle’s parameters. We filtered Sol fascicle length using a 4th-order low-pass Butterworth filter (10 Hz).

### Statistical Analyses

We averaged the value of each time series in 75 ms epochs, from 75 ms before the perturbation (baseline) to 225 ms after perturbation onset for each participant and condition. First, we used Shapiro–Wilk tests to verify the normal distribution of the data. Then, for each recorded signal at each time window, we performed a paired two-tailed *t* test to compare relaxed and coactivated conditions. We also used a paired *t* test to assess differences in step threshold between relaxed and coactivated conditions. A few variables were not normally distributed; in these cases, we confirmed our results using Wilcoxon rank-sum tests. We used linear regression to assess the relationship between the difference in step threshold and initial CoM position across the coactivated and relaxed conditions. Significance level was set at α = 0.05, adjusted with a Bonferroni correction for nine comparisons (α = 0.0056).

## RESULTS

**Figure 2.**
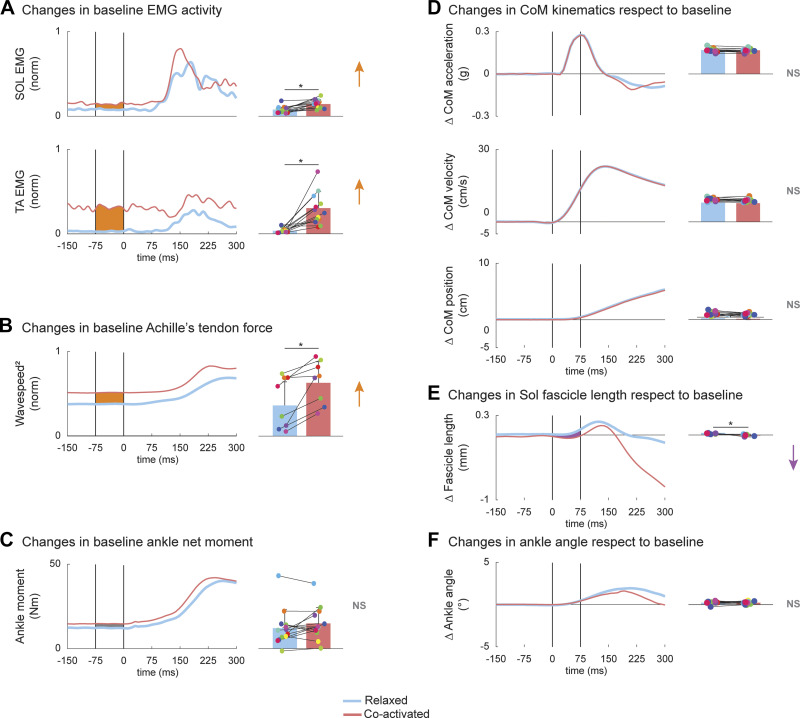
Changes in coactivation pre perturbation and its effects on the initial mechanical response. Averaged time-series across all participants (*n* = 13) for soleus (Sol) and tibialis anterior (TA) (*A*), squared wave speed (*n* = 8; *B*), ankle moment (*C*) during relaxed (blue) and coactivated (red) conditions. To differentiate the feedback response from feedforward strategy, baseline mean values (0–75 ms prior the perturbation) were subtracted to each averaged time-series across all participants (*n* = 13) for center of mass (CoM) kinematics (*D*), fascicle length (*n* = 6; *E*), and ankle angle (*F*) during relaxed (blue) and coactivated (red) conditions. Bar plots indicate means ± SD values for each signal for the relaxed (blue) and coactivated (red) conditions (right column) during the time window enclosed by the vertical lines (−75 to 0 ms for *A*, *B*, and *C*; 0–75 ms for *D*, *E*, and *F*). Each dot refers to a single participant (*n* = 13). The area and arrows in orange and purple describes significant increase and decrease during coactivated vs. relaxed trials. *Significant values (*P* < 0.05).

First, we validated our protocol. Providing visual feedback of Sol EMG activity and instructing participants to maintain their joint angles from the relaxed position, enabled participants to increase their feedforward muscle coactivation of Sol and TA. Although we only provided Sol visual feedback, all participants increased their TA activity (range 363% to 4,581% of increase from baseline) together with Sol (range 83% to 283% of increase from baseline) before each perturbation in coactivated trials versus relaxed trials. Both Sol and TA mean normalized activity were higher (Fig. 2*A*, *P* < 0.002) in coactivated (Sol 0.143 ± 0.044; TA 0.305 ± 0.191) with respect to relaxed trials (Sol 0.078 ± 0.051; TA 0.037 ± 0.038) during baseline (75 to 0 ms before perturbation onset). Two subjects were unable to maintain the same joint angle in relaxed and coactivated trials, resulting in a forward lean. However, our overall results remained the same when we repeated our analysis by excluding each subject who leaned forward. Therefore, we report the results from all trials.

The increase in feedforward muscle coactivation increased the tensile force acting on the Achilles tendon. We estimated this change in force via altered Achilles tendon shear wave velocities. Squared shear waves velocities were 73% faster during baseline in the coactivated versus relaxed trials (Fig. 2*B*, *P* < 0.007), indicating greater forces along the tendon (Fig. 2*B*). Due to minimal joint movements when people coactivated their leg muscles, baseline ankle joint moments computed using inverse dynamics were not different in the coactivated versus relaxed trials (Fig. 2*C*, *P* = 0.135).

In contrast with our initial hypothesis, increased feedforward muscle coactivation did not provide stronger resistance to external perturbations. CoM and ankle kinematics immediately following the perturbation were not different across relaxed and coativated conditions. The initial (0–75 ms after perturbation onset) mean CoM forward acceleration, velocity, and position were not different in coactivated versus relaxed trials (Fig. 2*D*, *P* > 0.157). In addition, the increase in feedforward coactivation did not change the initial ankle dorsiflexion in the coactivated versus relaxed conditions (Fig. 2*F*, *P* = 0.967). However, the mean initial stretch of the underlying Sol fascicle was reduced by ∼130% (Fig. 2*E*, *P* < 0.027) during coactivated with respect to relaxed trials (−0.011 ± 0.023 mm vs. 0.035 ± 0.011 mm, respectively).

**Figure 3.**
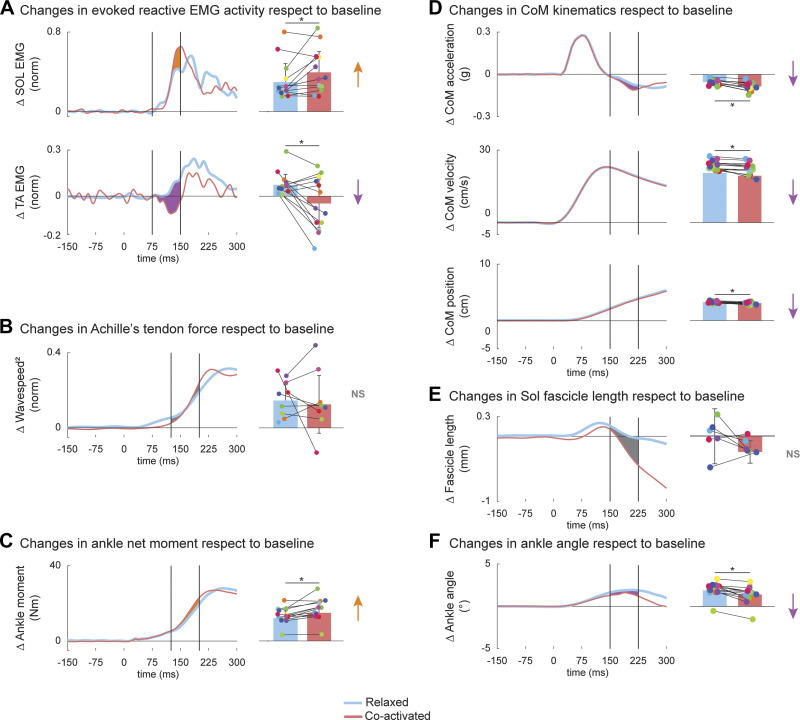
Changes in feedback response and its effects on the subsequent mechanical response. To differentiate the feedback response from feedforward strategy, baseline mean values (0–75 ms prior the perturbation) were subtracted to each averaged time-series across all participants (*n* = 13) for soleus (Sol) and tibialis anterior (TA) (*A*), squared wave speed (*n* = 8; *B*), ankle moment (*C*), center of mass (CoM) kinematics (*D*), fascicle length (*n* = 6; *E*), and ankle angle (*F*) during relaxed (blue) and coactivated (red) conditions. Bar plots indicate means ± SD values for each signal for the relaxed (blue) and coactivated (red) conditions (right column) during the time window enclosed by the vertical lines (75–150 ms for *A*; 125–200 ms for *B* and *C*; 150–225 ms for *D*, *E*, and *F*). Each dot refers to a single participant (*n* = 13). The area and arrows in orange and purple describes significant increase and decrease during coactivated vs. relaxed trials. *Significant values (*P* < 0.05).

The feedback response to the perturbation was characterized by a decrease in muscle coactivation, as shown by the increase in Sol and the reduction in TA initial burst versus baseline, leading to a faster net ankle plantar flexion torque generation. Despite a reduction in Sol initial fascicle stretch (Fig. 2*E*), mean Sol activity increased 32% with respect to baseline (Fig. 3*A*, *P* = 0.036) during the 75–150 ms time window after the perturbation during the coactivated versus relaxed trials. The increased Sol EMG initial burst did not affect squared shear wave velocity (Fig. 3*B*, *P* = 0.561) in the 125–200 ms time window after the perturbation. Alternatively, mean TA activity with respect to baseline was lower by ∼164% (Fig. 3*A*, *P* = 0.009) in the 75–150 ms time window (−0.047 ± 0.159 for coactivated, 0.073 ± 0.069 for relaxed). This increase in the reciprocal muscle activation increased net ankle torque by ∼23% (Fig. 3*C*, *P* = 0.004) with respect to baseline during the coactivated versus relaxed trials (14.9 ± 5.2 Nm vs. 12.1 ± 3.8 Nm, respectively). In turn, the faster net ankle moment production slightly reduced mean CoM forward acceleration (Fig. 3*D*, −0.085 ± 0.033 g for coactivated, −0.055 ± 0.02 g for relaxed, *P* = 0.0003), velocity (19.3 ± 2.2 cm/s, 20.4 ± 1.7 cm/s, *P* = 0.012), and displacement (3.1 ± 0.2 cm, 3.3 ± 0.18 cm, *P* = 0.041) with respect to baseline, during the 150–225 ms after the perturbation onset. In addition, increased feedforward coactivation reduced mean ankle dorsiflexion by 33% during the 150–225 ms (Fig. 3*F*, *P* = 0.029) in the coactivated versus relaxed conditions.

**Figure 4.**
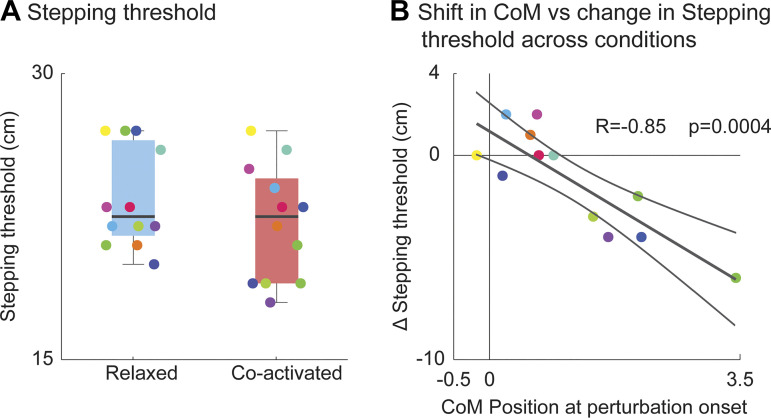
Effects of feedforward coactivation on balance capacity. We evaluated the individual step threshold (*n* = 12) during both relaxed and coactivated conditions (*A*). The linear regression model describes the association between the variation of step threshold (coactivated–relaxed) and the initial center of mass (CoM) position at perturbation onset (*B*). Each dot refers to a single participant (*n* = 12). On each box, the central mark indicates the median, and the bottom and top edges of the box indicate the 25th and 75th percentiles, respectively. The whiskers extend to the most extreme data points not considered outliers.

Despite the faster reactive torque with increased feedforward muscle coactivation, participants have better reactive balance capacity at greater perturbation magnitudes compared with the relaxed trials (*P* = 0.12). Compared with relaxed trials, feedforward coactivation reduced the step threshold in six participants and improved the step threshold for three participants, but did not change the step threshold for the remaining four participants (Fig. 4*A*). Notably, the difference between participant’s initial CoM position during coactivated and relaxed trials (with respect to their base of support) correlated with their step threshold (Fig. 4*B*, *R* = −0.85 *P* = 0.0004), indicating that participant balance capacity decreased when they leaned forward irrespective of muscle coactivation.

## DISCUSSION

This is the first study testing the hypothesis that feedforward muscle coactivation leads to increased coactivation during feedback muscle responses to balance perturbations. In contrast with our initial hypothesis, feedforward coactivation did not increase feedback muscle coactivation in healthy young adults, suggesting that feedforward and feedback coactivation may be mediated by independent neural mechanisms. Instead, we found that voluntary feedforward muscle coactivation enabled faster ankle torque generation by increasing reactive agonist muscle activation and decreasing reactive antagonist muscle activation. We further found that the body’s initial mechanical response to perturbation did not change with voluntary feedforward coactivation of leg muscles, and there was minimal change in the total body motion or step threshold. These results contrast with findings in older adults and the idea that muscle coactivation increases postural stiffness and mechanical stability in anticipation of postural perturbations (35). Our results suggest that voluntary muscle coactivation may be a strategy used in young adults for reducing the time to generate reactive joint torque, and may be used in different contexts than muscle coactivation intended to increase body stiffness during postural perturbations. As such, it remains unclear whether involuntary feedforward and feedback muscle coactivation seen in threatening or uncertain conditions, with aging and disease are mechanistically coupled and increase postural stiffness.

Our results suggest that in young adults, feedforward and feedback coactivation can be modulated by two independent neural mechanisms. Here, we refer specifically to muscle coactivation that is above and beyond that required by the musculoskeletal constraint of the task and refers to additional muscle activation operating with the redundant null space of a task (8, 9). Based on studies in the upper limb and in standing postural control we hypothesized that increased feedforward coactivation would cause an increase in feedback coactivation (24, 62, 63). Typically, concomitant feedforward coactivation and agonist-antagonist feedback coactivation are observed when participants are required to stabilize joints when encountering mechanical disturbances (62, 64, 65) or in the presence of a motor disease (18, 30). Common neural mechanisms, like increased spinal reflexes or central gains (66, 67) have been hypothesized to elicit coactivation in both feedforward and feedback control during challenging conditions or in the presence of a motor disease (68). In our study, however, participants were asked to voluntarily increase feedforward muscle coactivation using visual biofeedback, which may use different mechanisms of muscle coactivation. Our results show that the simple presence of feedforward muscle coactivation does not necessarily lead to increased dynamic coactivation during the feedback response.

Rather, increasing feedforward coactivation through visual biofeedback enabled faster joint torque generation (better agility) through reciprocal muscle control. This strategy consists of reactively increasing agonist muscle activity while decreasing the activity of the antagonist. Decreasing feedback antagonist muscle activity requires that the antagonist muscle is activated before the perturbation, as sizable reductions are only possible when background muscle activity is above baseline levels. Similarly, increasing feedforward coactivation has been shown to improve motor performance in upper limbs’ postural tasks following a disturbance by allowing reciprocal agonist-antagonist control (69). Reciprocal muscle control may also be a minimum-effort solution to stabilize posture in the presence of sensorimotor noise (70). Along with the inhibition of TA (antagonist muscle), we observed an increase in the initial burst of activity of the Sol (agonist), despite a reduction in its initial fascicle stretch. This increase could have been facilitated by the motorneuron being at a higher activation level (71), or due to higher muscle spindle sensory signals due to either increased α-γ coactivation (72–74), or to increased muscle short-range stiffness with increased feedforward muscle activation (75, 76). The simultaneous increase in Sol and decrease in TA activity enabled faster ankle torque generation. Acting faster may be fundamental in uncertain conditions (50), or when longer neural delays (77, 78) prevent a proper timing of the muscular response.

Although the common hypothesis on the role of feedforward coactivation is an increase in mechanical impedance (79, 80), this effect was not noticeable in our data. The initial mechanical response to a postural perturbation can be attributed to stabilizing torques due to the intrinsic properties of the musculoskeletal system despite minimal joint angle changes (46, 81). Active muscles behave like springs whose stiffness increases nonlinearly with activation (82–84), but their manifestation at the whole body level may not be obvious (85). In contrast to increased voluntary coactivation in a single upper limb joint (86, 87), we did not see a reduction in the body’s initial acceleration in the presence of feedforward ankle muscle coactivation, which depends on the modulation of multiple body segments.

The lack of change in balance capacity in our experiment is consistent with the fact that we did not elicit increased feedback coactivation. A previous study in older adults showed that higher feedback coactivation but not feedforward coactivation was correlated with postural task failure (34). But, despite faster reactive ankle torque, we also did not see improvement in balance capacity; this may be due to the step threshold being largely determined by a hip strategy rather than an ankle strategy (53, 88). Thus, participants may have been able to mitigate the effect of the changes in ankle muscle coactivation and obtain a similar performance with and without feedback coactivation. It remains to be seen whether feedback coactivation affects balance capacity in young adults.

This study has potential limitations that did not influence our overall conclusions. First, once the perturbation started, we could not assume that the same level of feedforward coactivation was maintained throughout the duration of the perturbation. A previous study showed that the relaxation time of voluntarily contracted Sol muscle in response to a visual stimulus varies from 200 to 350 ms, depending on the level of prior muscle contraction (58). Thus, we only analyzed the first 150 ms with respect to the platform onset, in which we believe the dynamic feedback response elicited by the perturbation to be summed with feedforward muscle activity. Yet, there is some evidence that long-latency responses do not start until 100 ms (89, 90). However, our analysis of 100–200 ms resulted in the same conclusions as the analysis of 75–150 ms. Next, to achieve and maintain the desired level of coactivation, some participants leaned forward before the perturbations. Although the initial postural configuration could theoretically influence the results, we did not find significant differences in the neuromechanical strategies adopted by the subjects who leaned forward, other than a decrease in step threshold. Also, although step threshold has been validated in several studies as a measure of balance capacity (91, 92), to identify balance impairments (51, 93) and to distinguish between fallers and non-fallers (94), its sensitivity may be population-specific and not able to capture subtle differences in balance capacity induced by coactivation. In addition, shear wave tensiometry captured the increase in tendon force due to increased muscle coactivation before the perturbation, but we were unable to estimate absolute Achilles tendon forces throughout our trials. The shear wave tensiometry demonstrated a relative increase in tendon force during coactivation that could not be inferred from inverse dynamics that only consider net joint torques (95). Although there is a linear relationship between squared wave speed and tendon force (59, 60), a subject-specific calibration procedure is necessary to obtain absolute values of tendon forces (96). Finally, the present study considered only the effect of coactivation of ankle muscles during backward platform translations. Thus, it must be acknowledged that different perturbation types (i.e., rotational) or directions may not benefit from muscle coactivation, and it may actually be destabilizing, thus our results may not be generalizable perturbation (10, 38, 40, 41).

Importantly, we need to make a distinction between the voluntary activation of muscles to achieve coactivation used in this study, and nonvoluntary activity that is likely underlying changes in coactivation observed in older adults, individuals with balance disorders, and/or challenging conditions. Indeed, several descending pathways can be involved in the origin of nonvoluntary muscle coactivation. Increased coactivation has been associated with basal ganglia (18) and cerebellar dysfunctions (97), and to spasticity of both spinal and supraspinal origin (22, 68). Thus, nonvoluntary coactivation induced by different pathways may instead increase feedback coactivation by, for example, reducing reciprocal inhibition. Furthermore, the increase in attentional demand associated with the biofeedback task may represent an additional confounding factor in the comparison of voluntary and nonvoluntary coactivation.

Our study suggests that there may be at least two different roles of feedforward muscle coactivation in responses to standing balance perturbations. Here, we show that voluntary coactivation could be used to increase agility by enabling a faster rate of rise in joint torque response by reciprocal agonist-antagonist neuromuscular control. Similar mechanisms are observed in animals that facilitate rapid movements by reducing antagonist muscle activity (98–100). In contrast, feedforward coactivation for stability enables faster mechanically mediated balance responses that may not require as precise sensorimotor feedback responses for balance (101, 102). It remains to be seen whether the mechanisms of feedforward muscle coactivation differ in these two circumstances, and in aging and disease. However, simple coactivation indices may not provide adequate information to understand the function of feedforward coactivation, since the context in which muscle coactivation is induced experimentally may be critical to the study outcomes. The effects of feedforward and feedback coactivation may also differ between the upper and lower limb tasks, based on whether the movement involves a closed or open kinematic chain (36, 103). In conclusion, feedforward coactivation may have different effects on feedback activation, and movement depends on whether the desired output is an increase in agility or stability.

## DATA AVAILABILITY

Data will be made available upon reasonable request.

## GRANTS

This study was supported by National Institutes of Health Grants F32 AG063460, R01 HD46922, R01 HD90642, and McCamish Parkinson’s Disease Innovation Program.

## DISCLOSURES

No conflicts of interest, financial or otherwise, are declared by the authors.

## AUTHOR CONTRIBUTIONS

G.M., O.N.B., and L.H.T. conceived and designed research; G.M. and O.N.B. performed experiments; G.M. analyzed data; G.M., O.N.B., and L.H.T. interpreted results of experiments; G.M. prepared figures; G.M. drafted manuscript; G.M., O.N.B., and L.H.T. edited and revised manuscript; G.M., O.N.B., and L.H.T. approved final version of manuscript.
